# Enoxacin and Epigallocatechin Gallate (EGCG) Act Synergistically to Inhibit the Growth of Cervical Cancer Cells in Culture

**DOI:** 10.3390/molecules24081580

**Published:** 2019-04-22

**Authors:** Anna Margaret McDonnell, Holly M. Pyles, Edgar S. Diaz-Cruz, Christopher E. Barton

**Affiliations:** 1 Department of Biology, Belmont University; 1900 Belmont Boulevard, Nashville, TN 37212, USA; annamargaret.mcdonnell@pop.belmont.edu (A.M.M.); holly.pyles@pop.belmont.edu (H.M.P.); 2 Department of Pharmaceutical Sciences, College of Pharmacy, Belmont University; 1900 Belmont Boulevard, Nashville, TN 37212, USA; edgar.diaz-cruz@belmont.edu

**Keywords:** oncology, cancer, apoptosis, cell cycle, HeLa, enoxacin, EGCG

## Abstract

Cervical cancer is a major cause of death in females worldwide. While survival rates have historically improved, there remains a continuous need to identify novel molecules that are effective against this disease. Here, we show that enoxacin, a drug most commonly used to treat a broad array of bacterial infections, is able to inhibit growth of the cervical cancer cells. Furthermore, our data show that epigallocatechin gallate (EGCG), a plant bioactive compound abundant in green tea, and known for its antioxidant effects, similarly functions as an antiproliferative agent. Most importantly, we provide evidence that EGCG functions synergistically against cancer cell proliferation in combined treatment with enoxacin. These data collectively suggest that enoxacin and EGCG may be useful treatment options for cases of cervical cancer.

## 1. Introduction

Cervical cancer is a leading cause of cancer death in women of all ages [1]. Recent predictions are that there will be nearly 13,000 women diagnosed with invasive cervical cancer this year [1]. Recent reports suggest that the majority of cervical cancers are associated with HPV infection [2]. Given the increasing rates of cervical cancer, it is imperative that research continues to focus on identifying novel molecules that may be effective at inhibiting the growth of cervical cancer cells.

Epigallocatechin gallate (EGCG) is a plant bioactive molecule showing promise as an anticancer agent, and is commonly found in green tea [3]. Previous research has shown that EGCG is capable of stopping the growth of numerous cancer cell types when used in culture [3,4]. More interesting are recent reports that EGCG is able to function synergistically with a number of commonly-used chemotherapeutic drugs [3,4]. Based on these studies, it seems reasonable that EGCG should be explored in combination with other anticancer drugs to determine whether it may function in a synergistic manner.

Enoxacin is a molecule displaying antibiotic properties [5]. In addition to its antibacterial function, enoxacin has also been shown to effectively inhibit the growth of multiple cancer cell types [6,7,8]. Interestingly, enoxacin has been reported to have a low incidence of toxicity due to its ability to selectively block the growth of cancer cells, while leaving noncancerous cells unaffected [9]. There is little evidence to suggest its efficacy against cervical cancer cells, suggesting a need for further studies in this area.

Herein, we hypothesized that enoxacin will act to block the growth of cervical cancer cells in culture. Secondly, we hypothesized that the plant bioactive, EGCG, may function synergistically with enoxacin in this setting. Our data suggest that enoxacin does, indeed, act to block the growth of cervical cancer cells in culture. Furthermore, we also show that EGCG acts synergistically with enoxacin in this in vitro model system.

## 2. Results

Given that enoxacin and EGCG have previously been shown to display anti-proliferative properties against some cancer cell types [6,7,8], we hypothesized that they may also inhibit cervical cancer cells grown in culture. HeLa cells were grown in culture and exposed to enoxacin or EGCG for varying amounts of time. 

**Figure 1:**
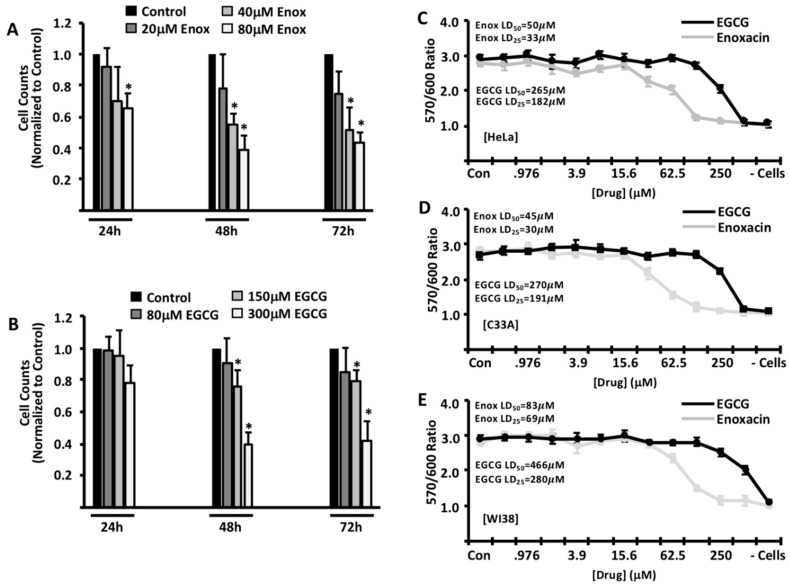
Effects of enoxacin and EGCG on cervical cancer cells in culture. HeLa cells were treated with a time course of varying doses of enoxacin (**A**) or EGCG (**B**) then counted to analyze cell numbers. Cell counts are presented relative to control (DMSO)-treated cells. HeLa (**C**), C33A (**D**) and WI-38 (**E**) cells were treated with serially-diluted concentrations of drug for 48 h and viability determined via the reduction of alamarBlue. (- cells) denotes alamarBlue reduction in the absence of plated cells. Data are representative of three independent experiments. LD_50_ values are denoted in each respective figure. * *p* < 0.05.

After exposure, cells were trypsinized and counted, and cell counts were normalized to control (DMSO) treated cells. While there was only a modest decrease in cell counts 24 h after exposure, there was a significant decrease in cell numbers that correlated with the concentration of enoxacin and EGCG used at 48 h (Figure 1A,B). Importantly, a further reduction in cell counts was not observed at 72 h, suggesting that the maximum effects of each drug are observed at the 48 h timepoint.

To validate these findings with another methodology, HeLa cells were exposed to each drug for 48 h, and viability measured via alamarBlue reduction (Figure 1C). Consistent with our previous data, both enoxacin (LD_50_ = 50 μM; LD_25_ = 33 μM) and EGCG (LD_50_ = 265 μM; LD_25_ = 182 μM) reduced the viability of HeLa cells, respectively. To determine whether these findings are specific only to HeLa cells, we carried out similar experiments with another cervical cancer cell line, C33A, as well as the normal human fibroblast cell line, WI-38. As shown in Figure 1D, C33A cells are similarly sensitive to both enoxacin (LD_50_ = 45 μM; LD_25_ = 30 μM) and EGCG (LD_50_ = 270 μM; LD_25_ = 191 μM), respectively. While normal human fibroblasts were sensitive to the effects of enoxacin (LD_50_ = 83 μM; LD_25_ = 69 μM), our data suggest that WI-38 cells are significantly less sensitive to the effects of EGCG (LD_50_ = 466 μM; LD_25_ = 280 μM) (Figure 1E)

**Figure 2:**
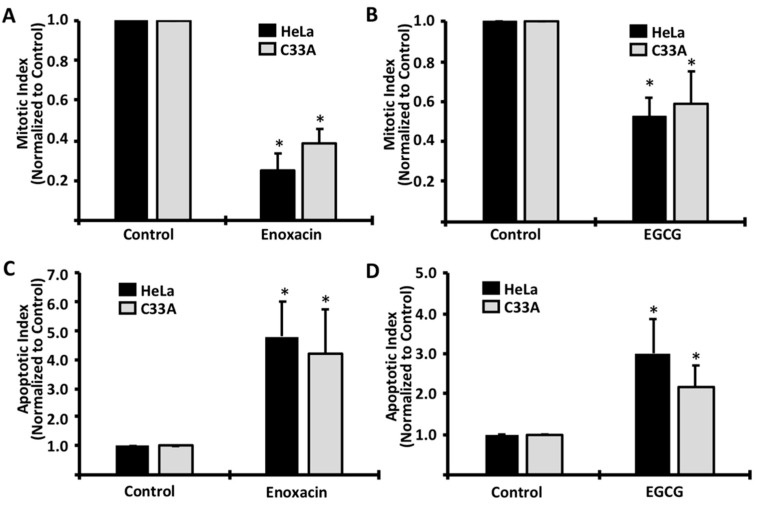
Enoxacin and EGCG induce cell cycle arrest and apoptosis in HeLa and C33A cells. Cells were exposed to LD_50_ concentrations of enoxacin (HeLa = 50 μM; C33A = 45 μM) or EGCG (HeLa = 265 μM; C33A = 270 μM) for 48 h and Hoechst staining was used to determine mitotic (**A**,**B**) and apoptotic (**C**,**D**) indices via nuclear morphology. Data are representative of three independent experiments. * *p* < 0.05.

To examine the mechanism driving the viability reduction, HeLa and C33A cells were treated with enoxacin or EGCG, and then stained with Hoechst in order to visualize nuclear components. This technique exposes distinct nuclear morphologies that allow direct observation of apoptotic and mitotic cells, and has previously been shown as an effective method for examining these two cellular processes [10]. In both cervical cell lines, we observed a significant decrease in mitotic cells following exposure to both enoxacin and EGCG (Figure 2A,B, respectively), suggesting that both enoxacin and EGCG were indeed functioning in an antiproliferative manner. Additionally, we observed a significant increase in cells displaying an apoptotic “blebbing” nuclear morphology, following exposure to both drugs (Figure 2C,D), suggesting that enoxacin and EGCG each induced apoptotic signaling pathways in both HeLa and C33A cells.

**Figure 3:**
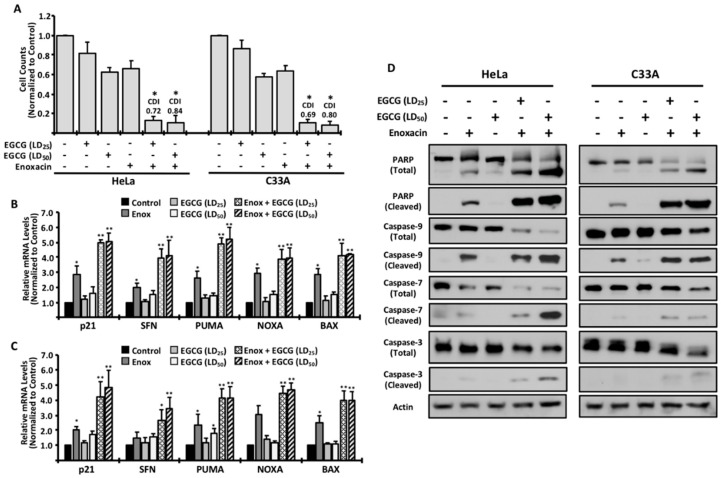
EGCG functions synergistically with enoxacin. Cells were treated as indicated and counted at 48 h (**A**). For analysis of synergism, the coefficient of drug interaction (CDI) is listed in (**A**) for co-treatments. Real-time PCR was used to examine gene expression of cell cycle-arrest and apoptotic genes in HeLa (**B**) and C33A (**C**) cells following the indicated treatments. All data are normalized to control-treated cells and are shown +/− standard deviation. * *p* < 0.05 relative to control-treated cells. ** *p* < 0.01, relative to control-treated cells. To analyze protein markers of apoptosis, cells were treated and Western blot was used to measure the levels of apoptosis-related proteins (**D**). All data are representative of three independent experiments.

Previous studies have reported that EGCG has the ability to function synergistically with commonly-used chemotherapeutic compounds [3,4]. Based on these studies, we treated both cell lines with enoxacin and different concentrations of EGCG, and measured the number of viable cells. Our data suggest that, while EGCG was able to moderately decrease the growth of both cells, there was a significantly greater effect when combined with enoxacin (Figure 3A). 

To determine whether the effects were synergistic in nature, we calculated the Coefficient of Drug Interaction (CDI), previously reported to be an effective, and widely-accepted, method of assessing synergism between two molecules [11]. When used to test synergism, a CDI below 1.0 suggests that two molecules function synergistically, with a CDI ≤ 0.7 indicating strong synergism. Co-treatment in HeLa cells revealed that EGCG at the LD_25_ concentration (CDI = 0.72) and LD_50_ concentration (CDI = 0.84) functioned synergistically when combined with enoxacin. Similar results were observed in C33A cotreatments with enoxacin and EGCG (LD_25_, CDI = 0.69; LD_50_, CDI = 0.80), suggesting that our findings are not specific only to HeLa cells. These data are further supported by quantitative gene expression analyses of cell cycle arrest and apoptotic genes when enoxacin is used in combination with EGCG. While single treatments resulted in a modest increase in gene expression, cotreatment with enoxacin and EGCG substantially increased transcript levels of all genes analyzed (Figure 3B,C).

Also in agreement with the CDI values shown in Figure 3B is the observation that combining enoxacin with LD_50_ concentrations of EGCG is effective at inducing the expression of arrest and apoptotic genes, but does not significantly increase the expression over LD_25_ values, again suggesting that lower concentrations of EGCG are sufficient to provide a synergistic effect with enoxacin (Figure 3B,C). 

To confirm these observations at the protein level, cells were treated and analyzed via Western blotting. While exposure to EGCG alone resulted in negligible apoptotic activity, as measured by Poly(ADP-ribose) Polymerase (PARP) cleavage and multiple cleaved caspase proteins (Figure 3D), cotreatment with enoxacin and EGCG resulted in cleaved PARP levels that were significantly higher than when treated with enoxacin alone, consistent with the synergism data shown in Figure 3A. Moreover, cleavage of multiple caspase proteins further support the idea that co-treatment with EGCG and enoxacin is more efficient at inducing an apoptotic cell death than with either drug in isolation (Figure 3D). Taken together, our findings support the hypothesis that EGCG, while perhaps not ideal as an isolated anticancer agent, functions synergistically when combined with additional antiproliferative agents such as enoxacin. 

## 3. Discussion

Cervical cancer is a major concern and currently stands as the fourth most common cause of death in females across the world [12]. It is currently thought that most cases of cervical cancer are the result of HPV [12]. Given the relatively low vaccination rate for HPV, cancer remains a common disease in females [2,13], thus it is critical researchers continue to identify novel molecules that are effective at stopping the growth of cervical cancer cells.

Enoxacin is a molecule that belongs to a larger class of fluoroquinone antibiotics [14]. Additionally, there is little evidence to suggest it displays significant toxicity towards humans [14]. Recently, a few reports have suggested that enoxacin may be effective at inhibiting the growth of prostate and lung cancer [6,7,8], as well as colorectal cancer cells [9]. Our data are the first to suggest that enoxacin functions as an effective anti-proliferative drug against cervical cancer cells in culture. We are currently unsure of the mechanism behind the cell cycle arrest and cellular death initiated by enoxacin. A previous report suggested that enoxacin is able to interfere with the function of DNA topoisomerases, enzymes that are critical for removing dangerous structures in DNA [15]. Given this potential mechanism of activity for enoxacin, it is plausible that enoxacin non-specifically targets normal WI-38 cells as well as the cervical cancer cells used in this study. Molecules that affect that activity of human topoisomerases (i.e., etoposide) certainly have growth-inhibitory properties against both cancer cells and normal cells, but have been used successfully in the clinic for a many years [16]. Future studies are necessary to confirm whether enoxacin is indeed actively targeting topoisomerases.

EGCG has been shown to have growth-inhibitory properties against a number of cancer cells both in vitro and in vivo [17], and is most often studied for its ability to act synergistically with other growth-inhibitory drugs [18]. In addition, EGCG is gaining interest clinically because it has also been reported to decrease a number of unwanted side effects that result from the use of chemotherapeutics (Wessner et al., 2007). Our data suggest that, while EGCG maintains a slight ability to negatively affect the growth of both HeLa and C33A cells in culture as a single agent, the concentration required to observe such an effect is, perhaps, unrealistically high. As a result, we propose that a more practical use for EGCG, based on previous literature and our findings, may be as a synergistic agent to increase the efficacy of other antiproliferative molecules. Our data suggest that, even when used at lower concentrations, EGCG is effective as a synergistic agent with enoxacin. The effectiveness of EGCG as a synergistic agent at these lower concentrations is further supported by gene expression and protein analyses presented here. Further research is needed to identify the molecules and/or pathways that mediate the synergistic nature of EGCG.

In closing, our data support synergistic activity between enoxacin and EGCG. Based on this study, we propose that enoxacin and EGCG be further analyzed as a potential treatment option for cervical cancers. Further research is currently being planned to extend upon these studies. 

## 4. Materials and Methods 

### 4.1. Cell lines and Culture Conditions

HeLa, C33A, and WI-38 cells were purchased from ATCC. Cells were cultured in monolayer in Dulbecco’s Modified Eagle Medium (DMEM; Gibco) supplemented with 10% heat-inactivated fetal bovine serum (Gibco) and 1% penicillin/streptomycin (Gibco).

### 4.2. Cell Proliferation and Viability Assays

Enoxacin (#94426) and EGCG (#4143) were purchased from Sigma-Aldrich, diluted in DMSO, and stored at 40C. For proliferation assays, 2.5 × 10^4^ cells were plated in 12-well plates and exposed to enoxacin or EGCG for 48 h. 48 h after treatment, cells were trypsinized and counted with a TC10 Automated Cell counter (BioRad) and cell numbers were normalized to control wells. For viability assays, 10,000 cells were seeded in triplicate in 96-well plates and incubated for 24 h. Medium was then replaced with either control medium or medium containing 2-fold dilutions of enoxacin or EGCG (starting concentration 500 μM) and incubated for 48 h. Viability was determined by the addition of alamarBlue for 4 h and reading at 570 nm. 600 nm readings were used to detect background signal and data were normalized by determing the ratio of 570 nm/600 nm.

### 4.3. Mitotic and Apoptotic Analyses

HeLa cells (2 × 10^4^) were grown on glass coverslips and exposed to enoxacin or EGCG for 48 h and mitotic and apoptotic analyses were carried out using Hoechst 33,342 as previously reported [10]. A minimum of five random images were taken from each slide and all cells on each image were counted and characterized. 

### 4.4. Gene Expression Analysis

Real-time PCR analyses were performed as previously reported [10]. Data from select genes were normalized to Actin expression and analyzed using the ΔΔCt method [19] for relative quantification.

### 4.5. Analysis of Drug Synergism

HeLa cells were treated with enoxacin, EGCG, or a combination of both for 48 h in culture. After that time, cells were counted to assay viable cells, and those raw counts were normalized to control-treated cells. To determine whether enoxacin and EGCG function synergistically, we calculated the Coefficient of Drug Interaction (CDI) using CDI = AB/(A × B), where AB is the normalized cell count for cells co-treated with both drugs, and (A x B) is the product of each single treatment group. With regards to the resulting value, a CDI < 1.0 suggests that two molecules function synergistically, a CDI ≤ 0.7 indicates strong synergism, and a CDI > 1 suggests antagonism, as previously reported [11].

### 4.6. Western Blotting Procedures

HeLa and C33A cells were treated with the indicated drugs and harvested. Cell pellets were lysed in RIPA buffer (Cell Signaling Technology) containing 1mM PMSF. Equal quantities of proteins (50 µg) were separated by 10% precast polyacrylamide gels and transferred onto nitrocellulose membranes. Membranes were incubated with the following primary antibodies: Cleaved PARP (Asp214) (D64E10), PARP, Cleaved Caspase-3 (Asp175) (5A1E), Caspase-3 (D3R6Y), Cleaved Caspase-9 (Asp330) (E5Z7N), Caspase-9 (C9), Cleaved Caspase-7 (Asp198) (D6H1), Caspase-7 (D2Q3L) (Cell Signaling Technologies); and β-Actin (I-19) (Santa Cruz Biotechnology) and further analyzed following the manufacturer’s recommendations.
